# Adherence to standard nursing protocols on nasogastric tube feeding in a secondary referral hospital in Ghana: comparing self-ratings by professional and auxiliary nurses

**DOI:** 10.1186/s12913-019-3931-6

**Published:** 2019-02-13

**Authors:** Robert Kaba Alhassan, Richard Tsikata, Richard Naatu Tizaawaw, Prince Asante Tannor, Perpetual Praba Quaw, Cecilia Aba Ata Awortwi, Martin Amogre Ayanore, Agani Afaya, Solomon Mohammed Salia, Japiong Milipaak, Prudence Portia Mwini-Nyaledzigbor

**Affiliations:** 1 Department of Public Health Nursing, School of Nursing and Midwifery, University of Health and Allied Sciences, Ho. PMB 31, Volta Region, Ho, Ghana; 2 Department of Nursing, School of Nursing and Midwifery, University of Health and Allied Sciences, Ho, Ghana; 3 School of Public Health, University of Health and Allied Sciences, Hohoe, Ghana; 4 Department of Midwifery, School of Nurising and Midwifery, University of Health and Allied Sciences, Ho, Ghana

**Keywords:** Adherence, Auxiliary nurses, Nursing practice, Ghana, Nasogastric (NG) tube feeding, Professional nurses, Secondary referral hospital, Standard nursing protocols

## Abstract

**Background:**

Data on nurses’ adherence to standard protocol on nasogastric (NG) tube feeding remain scanty in Ghana even though patients in critical medical conditions are routinely managed using this procedure. This study explored self-rated adherence to standard protocols on NG tube feeding among professional and auxiliary nurses and the perceived barriers impeding compliance to these standard protocols.

**Methods:**

This is a descriptive analytical cross-sectional study among professional (*n* = 89) and auxiliary (*n* = 24) nurses in a major referral hospital in one of the ten administrative regions in Ghana. Four-point Likert scale was used to ascertain the level of adherence to standard guidelines on nasogastric tube, ranging from 4 “Very large extent” to 1 “Very little extent”. Wilcoxon Mann-Whitney test and univariate ordered logistic regression tests (proportional odds models) were performed to determine the odds of higher self-ratings among professional and auxiliary nurses.

**Results:**

Overall, the odds of higher self-ratings on adherence to standard nursing protocols on NG tube feeding was higher among auxiliary nurses than professional nurses (OR = 2.76, *p* = 0.031) after adjusting for age, gender, education and years of work experience. Key barriers to adherence to standard protocols on NG tube feeding were: limited opportunities for in-service trainings and insufficiency of NG tube feeding protocols on the wards.

**Conclusion:**

There is the need for more routine in-service trainings for nursing staff to update their knowledge on NG tube feeding. Hospital management should also make current nursing protocols available to nurses to guide their practice alongside routine onsite supervision of nurses.

**Electronic supplementary material:**

The online version of this article (10.1186/s12913-019-3931-6) contains supplementary material, which is available to authorized users.

## Background

Feeding in order to meet the body’s dietary and physiological needs is a vital component of human survival [1]. In critical medical conditions, patients are unable to feed as they ought to due to a weakened state or other pathological conditions. Moreover, conditions such as pharyngeal tumours block passage of food into the gastrointestinal tract thus demanding feeding through NG tube. Likewise, neck injuries and a state of partial or complete unconsciousness (coma) are indications for nasogastric (NG) tube feeding.

To maintain patients’ physiological and nutritional status, assisted feeding in the form NG tube feeding is often the option and nurses play a critical role in the management of patients on this kind of medical therapy [2]. NG tube feeding, which is a type of enteral tube feeding, involves the delivery of nutritionally complete feed via a tube into a gut [2]. This feeding approach is used for patient who are unable to meet their nutritional requirements orally due to reasons stated earlier. In the medical literature different types of enteral routes of feedings have been documented which include: nasogastric, nasoduodenal, nasojejunal, oesoghaostomy, gastrostomy and jejunostomy routes. This study focused mainly on nasogastric route of feeding because it is commonly used in Ghana.

According to Delaune & Ladner [3], nasoenteral insertion of a gastric feeding tube is the simplest and most often used method of tube feeding. It has been found that enteral route of feeding has the advantage of helping in digestive system function; enteral feeding is also cheap and has more nutritional benefits to the patients compared to parenteral nutrition [4].

Nasogastric tube feeding is the most frequently used method of enteral feeding particularly if feeding is to be used for relatively shorter period. In view of this, the nurses’ knowledge and skill on the insertion of nasogastric tube and subsequent care are important to ensure patient safety [5, 6].

Adherence to standard protocols in the management of NG tube feeding is particularly critical in resource poor settings in Africa where quality of healthcare delivery and safety remain significant challenges [7, 8]. El-Meanawi [9] in a study in Egypt revealed that, 62% of nurses had unsatisfactory knowledge on nasogastric tube feeding. According to El-Meanawi [9] nurses’ knowledge and practices regarding nasogastric tube feeding at the medical and surgical departments were not enough with some unsafe practices [9].

In Ghana, professionals and auxiliaries nurses  are taught the standard nursing protocol for NG tube feeding as per the Nursing and Midwifery Council (NMC) curriculum for nursing trainees. Professional nurses are further taught the procedure for insertion of the NG tube and are permitted to perform this procedure especially in rural areas where there are no medical doctors to perform this role. On the other hand, auxiliary nurses (also called nurse-assistants clinical (NAC) and nurse-assitants preventive (NAP)) are basically supposed to assist professional nurses in NG tube insertion and its management. However, the reality is that in Ghana, due to human resource constraints, auxiliary nurses often outstep their mandate to perform these relatively advanced nursing procedures.

There is currently paucity of empirical data on NG tube feeding and related challenges at clinical settings in Ghana. Moreover, adherence to standard protocols in NG tube feeding has not been explored from the perspectives of nursing staff who play a critical role in NG tube feeding, hence the need for this study. This study thus explored self-rated adherence levels to standards on NG tube feeding by professional and auxiliary nurses and the related barriers impeding compliance with these standard protocols.

## Methods

### Research design, population and setting

This is a descriptive analytical cross-sectional study which employed quantitative data collection approach. The study was carried out at a major regional referral hospital in the Volta region, one of the ten (10) administrative regions in Ghana. The hospital also serves as a referral facility for neighbouring Togo. According to available administrative records, the hospital had^s^ a total staff population of 661 as at 2018. This number includes 262 nursing staff, 31 medical officers, 7 physician assistant anaesthetists, 2 physician assistant medical herbalists, 2 physician assistant medical staffs, 271 paramedics, 76 casual staffs and 10 health aids. The 262 nursing staff comprise of midwives (69), mental health nurses (11), community health nurses (16), registered general nurses (113) and enrolled nurses (53). The hospital has a bed capacity of 206 with a weekly Out Patient Department (OPD) attendance of about 800 to 950 patients.

### Sampling method

This study employed both probability and non-probability sampling techniques, namely, stratification of the hospital into strata of wards and convenience sampling for the nurses working within these strata (wards). Stratification was employed because the hospital had various units with unique features. Convenience sampling was used because of the varied work schedules of nurses which would not favour simple random or other forms of probability sampling approaches. Thus, the questionnaires were administered to respondents who met the inclusion criteria and were readily available to participate in the study.

The authors however, acknowledge that intra-respondent variances (design effect) within the two categories of nurses (professional and auxiliary) were potentially minimal which favoured the study design since the objective was to compare between the two different professional categories.

### Sampling and sample size determination

A total of eight (8) wards were purposively selected to form the strata from which respondents were sampled. The eight (8) wards selected were wards that managed patients on NG tube in one way or the other. Wards excluded did not have anything to do patients on NG tube feeding. A total sample size of 120 was considered based on Krejcie and Morgan [10] statistical formula^1^ for sample size determination based on known populations at 95% confidence level. The sample size was proportionally allocated to each of the eight (8) wards. Thus, approximately 15 professional and auxiliary nurses were sampled from each ward and because professional nurses constituted 68% of the total nursing staff, 68% of the 15 assigned quota was further allocated to professional nurses (*n* = 10) and the remaining 32% assigned to auxiliary nurses (*n* = 5).

### Inclusion/exclusion criteria

The study included only nursing staff (professional and auxiliary) who at the time of conducting the study had valid Nursing and Midwifery Council (NMC) of Ghana certification (i.e. Professional Identification Number (PIN) or Auxiliary Identification Number (AIN)). Another eligibility criterion was that the participant should have been a full time employee of the hospital; also, staff should have managed a patient on NG-tube at least in the last six months to promote relevance of staff responses on NG tube feeding. Student nurses, rotation nurses and part time nurses were equally excluded from the study.

### Data collection instruments and procedure

Field data was collected using a structured questionnaires that comprised of three (3) main sections: Section A (Socio-demographics and work history of staff), Section B (Adherence to standard practices in NG tube feeding) and Section C (Perceived barriers to standard practices in NG tube feeding). Questionnaire design was guided by the NMC standard protocol on NG tube feeding and the formats were closed and open ended.

The closed ended questions included Likert scale items ranked on the extent to which respondents adhere to the pre-determined standard procedures in NG tube feeding on a four-point scale of 4= “Very large extent” 3 “Large extent” 2 “Little extent” 1 “Very little extent” (see Additional file 1_Questionnaire). Cronbach’s alpha was checked for the Likert scale items and the scale reliability coefficient found to be 0.80 which is above the 0.70 rule of thumb according to Bleda and Tobias [11]; moreover, average inter-item covariance was 0.13.

Five (5) trained student researchers collected the data over a period of about ten (10) days in March, 2018. Questionnaires were administered randomly to eligible respondents who were available and willing to respond. Participants were assisted in the questionnaires administration; questionnaires were collected same day or a day after in some  instances  to promote high response rate.

Written informed consent was attained voluntarily from all staff prior to their participation, in line with the University of Health and Allied Sciences (UHAS), Research Ethics Committee (REC) guidelines for studies involving human subjects. Each questionnaire was administered over twenty-five (25) min approximately. In all, 120 questionnaires were administered and 113 were retrieved with complete data, representing a return rate of 94%. The final sample size used for the analysis did not include the 6% non-response rate; no missing data were recorded during the data analysis.

### Validity and reliability

Validity and reliability of the study instrument were determined through a review of relevant literature on standard protocols in NG tube feeding. Furthermore, the questionnaires content was validated by comparing the various items with the NMC, Ghana standard procedures for NG tube feeding [12]. The data collection instrument was pre-tested among twelve (12) different categories of nursing staff in a comparable hospital within the Ho municipality where the study was conducted. The pre-test enabled the researchers to correct ambiguity in the questionnaires, monitor average time for the questionnaires administration and typographical mistakes.

### Data analysis and processing

Field data were first captured into a Microsoft Excel Spreadsheet (2016), cleaned, coded and later exported to the STATA statistical software for data analysis (version 12.0 StataCorp, College Station. Texas USA). The 113 valid data were further disaggregated into two major sub-samples of professional (*n* = 89) and auxiliary (*n* = 24) nurses. Pearson chi square (*X*^2^) and Fisher’s exact tests were conducted as appropriate to ascertain the statistical differences between two sub-samples in terms of gender, religion, marital status, educational qualification, age and cumulative years of work experience.

Also, Wilcoxon Mann-Whitney test was used to determine differences among professional and auxiliary nurses in ten (10) key Likert scale items on standard practices in NG tube feeding. Mean scores were computed on the ten (10) point Likert scale where higher mean scores suggested better adherence and lower mean scores depicted lesser adherence by respondents. Overall adherence was computed as the ultimate dependent variable of interest (rank ordered 4=“Very large extent” 3 = “Large extent” 2 = “Little extent” 1=“Very little extent”). Seven independent variables were used to predict staff probability of adherence using a Univariate Ordered Logistic Regression (OLR) (proportional odds model). The main independent variable of interest was professional category (2 = professional nurse, 1 = Auxiliary nurse). Other variables controlled for in the regression were: gender, religion, marital status, educational qualification, age, years of work experience and overall experience on NG tube feeding. The regression output was reported in proportional odds ratio instead of coefficient values.

Multicollinearity diagnostics was conducted on all the independent variables of interest and the average Variance Inflation Factor (VIF) on the independent variables of interest was found to be 1.66. Moreover, none of the independent variables fitted into the regression models had VIF up to 10, the rule of thumb for exclusion [11]. All statistical tests for significance was determined at 95% confidence level.

## Results

### Socio-demographic data

Out of the 113 respondents who fully completed the questionnaires, 89 were professional nurses representing 79% and rest were auxiliary nurses. A greater proportion of the female distribution was among auxiliary nurses (83%) as compared to 53% in the case of professional nurses (*p* = 0.007); also, a greater proportion of the professional nurses were found to have higher educational qualifications than auxiliary nurses (*p* < 0.001). The average age of respondents was 29 ± 6. Professional nurses were averagely older (mean = 30 ± 5) than their auxiliary counterparts (mean = 29 ± 2). Professional nurses also worked for more years (mean = 5 ± 3) than auxiliary nurses (mean = 4 ± 2) (see Table 1).

**Table 1:** Demographic Characteristics of Respondents (*n* = 113)

Characteristics	Aux. Nurse (*n* = 24)	Prof. Nurse (*n* = 89)	Total (*n* = 113)	*p*-value
Gender	f (%)	f (%)	f (%)^*^	
Male	4 (17)	42 (47)	46 (41)	0.007^c^
Female	20 (83)	47 (53)	67 (59)	
Religion				
Christian	24 (100)	76 (86%)	100 (88)	0.155^a^
Muslim	0 (0)	11 (12)	11 (10)	
Others	0 (0)	2 (2)	2 (2)	
Marital status				
Single	12 (50)	47 (53)	59 (52)	0.494^b^
Married	12 (50)	42 (47)	54 (48)	
Educational qualification				
Certificate	23 (96)	2 (2)	24 (21)	0.000^a^
Diploma	1 (4)	49 (55)	51 (45)	
Degree	0 (0)	38 (43)	38 (34)	
Summary statistics	Mean (SD)	Mean (SD)	Mean (SD)	p-value
Age	29 (2)	30 (5)	29 (6)	0.384^d^
Work experience (years)	4 (2)	5 (3)	5 (3)	0.642^d^

Source: Field Data (2018); Legend: f (frequency); % *percentage); n (number of valid responses); *All frequencies are rounded to the nearest decimal; ^a^Fisher’s exact; ^b^1-sided Fisher’s exact; ^c^Chi-square test at 95% confidence level; ^d^Two-sample independent Student t-test at 95% confidence level

### Continuous professional training/education on NG tube feeding in the last one year

**Fig. 1:**
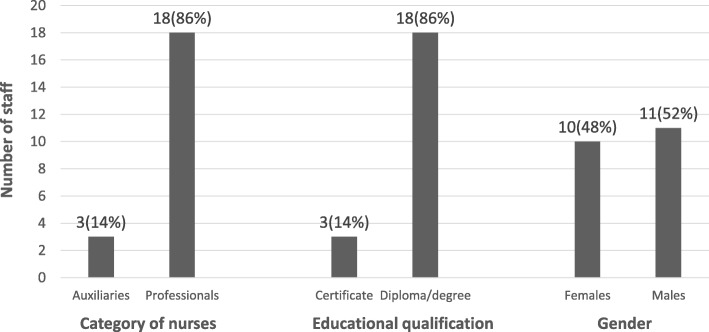
Staff who had continuous professional training/education on NG tube feeding in the last one year (*n* = 21). Source: Field Data (2018); Legend: NG (nasogastric)

It was found that out of the 113 staff interviewed only 21 (19%) said they had a continuous professional training or education on NG tube feeding in the last one year. Out of the 21 staff who have had the training 86% were professional nurses who either had a diploma or degree educational qualification. Also, more males (52%) than females (48%) had continuous professional training. The least category of staff who had a professional training were auxiliary nurses (14%) who also had certificate educational qualification (see Fig. 1).

### Adherence to standard protocols in NG tube feeding

Overall, auxiliary nurses self-rated themselves higher in terms of adherence to NG tube feeding standard protocol (mean = 1.60 ± 0.34) relative to professional nurses (mean = 1.39 ± 0.40), *p* = 0.005. The high self-rated scores were noticed in the areas of “explaining procedure to patient prior to feeding”; “checking the temperature of the feed prior to feeding”; “raising NG syringe for gravity during feeding”, and “immediate spigot of NG tube after feeding” (see Table 2).

**Table 2:** Self-ratings on adherence to standard nursing protocols by professional and auxiliary nurses (*n* = 113)

Likert scale items on NG tube feeding	Aux. Nurse	Prof. Nurse		*p*-value
	Mean^a^ (SD^b^)	Mean (SD)	Z	
Explanation of procedure to patient	1.38 (0.49)	1.16 (0.37)	2.34	0.019*
Upright position during intubation	1.63 (0.49)	1.45 (0.77)	2.16	0.031*
Lubrication of the tip of NG tube	1.38 (0.58)	1.19 (0.40)	1.57	0.118
Observation of patient mouth	1.58 (0.58)	1.38 (0.73)	2.18	0.030*
Aspirating of the gastric content	1.54 (0.51)	1.43 (0.74)	1.64	0.101
Pinching of kinking tube	1.71 (0.62)	1.56 (0.85)	1.61	0.108
10-15mls of water used to flush tube	1.67 (0.70)	1.63 (0.86)	0.74	0.462
Checking the temperature of feed	1.71 (0.81)	1.37 (0.53)	1.98	0.048*
Immediate spigot of NG tube	1.83 (0.82)	1.47 (0.66)	2.07	0.038*
Syringe for feeding is raised for gravity	1.63 (0.58)	1.24 (0.45)	3.40	0.001*
Overall practice adherence	1.60 (0.34)	1.39 (0.40)	2.80	0.005*

Source: Field Data (2017); Legend: *Wilcoxon rank-sum (Mann-Whitney) test, two-tailed test of hypothesis statistically significant at 95% confidence level; ^a^Mean values depict average adherence from summated four-point Likert scale of 4 = “Very large extent” 3 = “Large extent” 2 = “Little extent” 1 = “Very little extent”, thus higher mean scores depict better adherence to standard procedures in NG tube feeding and lower means suggest otherwise; ^b^SD (Standard deviation)

It was observed that the odds of adherence to standard NG tube feeding procedures is 2.76 times higher among auxiliary nurses than professional nurses (OR = 2.76, *p* = 0.031). Also, it was found that staff who were not married had higher odds of self-rating their adherence to standard protocols on NG tube feeding than married staff (OR = 5.68, *p* < 0.001).

Likewise, staff with more years of work experience had higher odds of rating themselves higher on adherence to NG tube feeding protocols than nurses with less years of work experience (OR = 1.25, *p* = 0.042). Similarly, respondents who appeared to have better experience with NG tube feeding (i.e. attending workshops on NG tube feeding) had greater odds of rating themselves higher on adherence than those with less or no experience on NG tube feeding (OR = 98.45, p < 0.001). Factors such as religion, gender, age and educational qualification did not significantly predict overall likelihood of higher self-ratings (see Table 3).

**Table 3:** Determinants of nurses’ self-ratings on adherence to standard practices in NG tube feeding (*n* = 113)

Independent variables	Dependent variables		
	Overall self-rated adherence		
	OR^+^(SE)	[95%Conf. Intv.]	*p*-value
Profession			
Auxiliary	2.76 (1.30)	[1.09 6.96]	0.031^++^
Professional	Ref	Ref	Ref
Gender			
Female	1.51 (0.59)	[0.70 3.24]	0.289
Male	Ref	Ref	Ref
Religion			
Christian	0.64 (0.43)	[0.17 2.39]	0.505
Other religions	Ref	Ref	Ref
Marital status			
Single	5.68 (2.29)	[2.58 12.51]	0.000^++^
Married	Ref	Ref	Ref
Education			
Degree	0.45 (0.18)	[0.20 0.99]	0.048
Other qualifications	Ref	Ref	Ref
Age	0.91 (0.06)	[0.80 1.04]	0.164
Work experience	1.25 (0.14)	[1.01 1.54]	0.042^++^
Overall experience on NGTF	98.45 (124.71)	[8.221178.86]	0.000^++^

Source: Field Data (2018); Legend: Ref (Reference dummy variable); Univariate Ordered logistic Regression (OLR); OR^+^ (Proportional Odds Ratio); Regression model: Log likelihood = − 237.60159; Number of obs = 113; LR chi2(8) = 49.37; Prob > chi2 = 0.0000; Pseudo R2 = 0.0941; ^++^Ordered logistic regression (OLR) test statistically significant at 95% confidence level

### Barriers to standard NG tube feeding practices

It was found that inadequate number of displayed NG tube feeding protocols was mentioned by 78 respondents as a key constraint to following the standard protocol on NG tube feeding; 103(91%) respondents identified lack of continuous professional development (CPD) trainings as a major limitation. Inadequate number of nurses per shift was another important barrier mentioned by 84(74%) respondents.

**Fig. 2:**
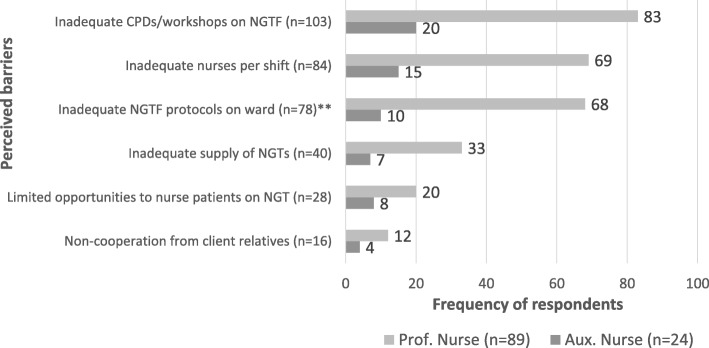
Perceived barriers to standard NG tube feeding practices. Source: Field Data (2017); ^**^1-Sided Fisher’s Exact (*p* = 0.002); Legend: NGTF (Nasogastric Tube Feeding); NGTs (Nasogastric Tubes); Aux. nurses (Auxiliary nurses); Prof. nurses (Professional nurses)

Out of the 113 respondents, 40(35%) said inadequate supply of the re-requisite NG tubes posed a challenge to adhering to standard practices. Limited opportunities to care for patients on NG tube was another important constraint mentioned by respondents 28(25%) while 16(14%) mentioned opposition from relatives of patients. The question on relatives’ opposition to NG tube feedings was deemed relevant in the context of this study because under the Patients Charter in Ghana, patients' relatives have the right to participate in the medical decisions and treatment options for patients. Figure 2 shows details of respondents’ perceived constraints to adhering to standard nursing protocols on NG tube feeding.

On the whole, the findings showed that self-ratings on adherence to standard nursing protocols were lower among professional nurses than their auxiliary counterparts who apparently had less exposure to workshops on NG tube feeding and other related career development opportunities (see Fig. 2).

## Discussion

Overall, this study explored the perspectives of auxiliary and professional nurses on the extent to which they adhere to standard nursing protocols on NG tube feeding and the possible barriers that constrain them in their efforts to adhere to these standard practices. Seven (7) broad standard components consistent with the Nursing and Midwifery Council (NMC) of Ghana standard operating procedures (SOPs) on NG tube feeding were used to assess extent of adherence by the sampled nursing staff. These standard criteria were then scored on a four-point Likert scale on the extent to which nurses think they adhere to them from 4 = “Very large extent” to 1 “Very low extent”. Thus, high summated mean scores suggest better adherence by staff and *vice-versa*.

Even though a number of studies have been conducted on NG tube feeding in the reviewed literature [9, 13–16], there remain dearth of literature on the comparative differences in adherence by professional and auxiliary nurses particularly within the context of low resourced countries such as Ghana. Moreover, in many previous studies the emphasis has been limited to knowledge levels and skills of clinicians on NG tube feeding [17] without a further exploration on the extent to which these clinicians adhere to the standards they claim to know.

On the whole, this study discovered that the extent to which nurses perceived their adherence to the standard nursing protocols on NG tube feeding was largely low. Counter intuitively, professional nurses appeared to score even lower self-ratings on adherence than their auxiliary counterparts (*p* = 0.005). A univariate ordered logistic regression test further confirmed the differences (OR = 2.76, *p* = 0.031). This revelation either suggests perhaps professional nurses were more honest in their responses as compared to auxiliary nurses or indeed the professional nurse knew the standards but for one reason or the other they are unable to follow them to the latter on routine basis. Also, perhaps because auxiliary nurses usually have relatively shorter and less detailed training in advanced nursing procedures such as NG tube feeding they might have displayed ignorance by rating themselves higher on practices that are below the standard practice.

Care rendered by professional nurses relative to auxiliary nurses has been found to significantly associate with the odds of better patient care [18]. A study by Aiken et al. [18] involving adult acute care hospitals in Belgium, England, Finland, Ireland, Spain and Switzerland concluded that a reduction in the proportion of professional nurses taking care of patients is associated with a 11% increase in the odds of death while substituting one nurse assistant for a professional nurse for every 25 patients is associated with a 21% increase in the odds of dying among these patients.

This empirical revelation, albeit not contextualized in Ghana, is compelling and raises the question whether or not the trend is relevant to the Ghanaian context where the number of auxiliary or assistant nurses outnumber professional nurses in many healthcare facilities in Ghana. As at 2016 a total of 13,239 registered general nurses (i.e. professional nurses) worked within the public health sector compared to 18,107 enrolled nurse (i.e. auxiliary nurses) [19].

Since this current study did not independently verify the self-ratings by the staff it remains a challenge to conclude that indeed professionals necessarily did not follow standard practices in NG tube feeding relative to their auxiliary colleagues. Perhaps the auxiliary nurses did not answer the questions honestly due to the bias of social desirability.

In addition, these results could be attributed to the fact in many resource poor health systems due to inadequate numbers of professional nurses, auxiliary nurses turn to assume the roles and duties of professional nurses and over time develop better competencies and proficiencies as alluded to in the literature [20]. Future researchers are therefore encouraged to explore beyond these subjective reports with an independent verification of staff self-ratings through onsite observations and scoring over time. Until this independent assessment is done findings of this current study remain subjective self-ratings by participants.

Apart from the professional category of respondents, factors such as number of years of work experience, exposure to NG tube feeding training workshops and marital status were found to be important predictors of nurses’ self-ratings on their adherence to standard nursing protocols on NG tube feeding. Similar studies in the past have alluded to the relevance of continuous professional development trainings towards improving professional practice at clinical sites [7, 21, 22]. For instance, a study by Bloomer et al. [20] found that years of clinical experiences has a positive correlation with the tendency to follow standard protocols in clinical practice and render better quality care. Higher self-ratings on adherence was observed among single staff than those who were married. This observation cannot immediately be explained even though the suspicion is that perhaps staff who are single are more likely to have relatively less family responsibilities since a positive association between family and work life has been implied in previous studies [7, 23, 24]. Gender, age, religion and educational qualification did not significantly influence adherence to standard nursing protocols on NG tube feeding contrary to similar previous studies [7, 23, 24] where these factors were strong predictive factors. Perhaps differences in study designs, settings and respondents accounted for these contrasting findings with some of the literature.

In terms of the barriers constraining adherence, lack of in-service training on NG tube feeding was the cogent constraint identified by 91% of the respondents. This constrain has been reported in similar studies on Ghana [7, 21, 25–28] and other countries [6, 17, 29]. Limited health budgets to support staff in this regard has culminated into lower frequencies of trainings for staff resulting in many clinicians practising with obsolete knowledge. Beyond the limited in-service trainings, deficits in health sector human resources remains a major challenge for many resource poor countries in Africa including Ghana [19, 30]. Unfortunately, these constraints continue to militate against quality of health service delivery by health staff including their ability to adhere fully to standard clinical practice as demonstrated in this study.

Inadequate number of nurses per work shift was identified by respondents as a prime challenge in adhering to standard protocols on NG tube feeding, similar to previous findings [6, 7, 25, 31]. As at 2016 the total number of health sector workforce was 102,019 serving close to 30 million Ghanaians [19]. In Ghana, the nurse patient ratio, at the time of conducting this study, was 1: 627 [32], and in the Volta region (where this study was conducted) the nurse patient ratio was 1:674 marginally above  the national average. Available administrative records from the study hospital also confirmed the nurses’ claim of low staff strengths given that there were a total of 166 nursing personnel serving an average OPD attendance of about 900 [33]. On the whole, the findings of this study suggest the need to address existing shortage of  nurses as part of efforts towards improving adherence to standard nursing practices and overall quality of care rendered to patients.

### Limitations

This study focused on one health facility from one (1) out of the ten (10) administrative regions in Ghana, thus creating generalizability challenges. Also, the responses were largely self-reported which could have introduced subjectivity into the study. In light of this, future researchers should adopt more objective approaches (i.e. independent scoring of staff performance) to minimize bias.

## Conclusion

Overall, self-rated adherence to standard nursing protocols on NG tube feeding by the sampled nursing staff was low. Self-rated adherence to pertinent standard nursing protocols was relatively lower among professional nurses than auxiliary nurses even though the former had higher educational qualification, more years of work experience and benefited more from workshops on NG tube feeding. These dynamics perhaps explain why professional nurses perceived their current practices as sub-standard. However, because these responses were largely subjective self-ratings, interpretation of the findings should be done with caution.

The major constraints identified by nurses regarding adherence to standard protocols on NG tube feeding were lack of routine workshops and trainings on NG tube feeding; shortage of nurses per shift; inadequate NG tube feeding protocols on the wards; inadequate supply of NG tubes and limited opportunities to care for patients on NG tube feeding.

In summary, findings from this study suggest interventions should be geared towards routine in-service trainings for nursing staff on NG tube feeding, and displaying more protocols and guidelines on NG tube feeding in relevant wards. In addition, there is the need to revisit activities of professional and auxiliary nurses on the wards to ascertain the real dynamics of task shifting among nursing staff.

### Implications for policy and clinical practice

Based on findings of this study the following recommendations are proposed to health managers and clinicians towards enhancing compliance to standard nursing protocols on NG tube feeding:

1. Intensify efforts on in-service training on new standard protocols for NG tube feeding, especially for staff working in medical-surgical wards and intensive care units where these procedures are more prevalent
2. Onsite clinical supervision of staff by senior nursing management should be intensified to ensure task sifting by professionals does not result in spillover effects and relegation of core duties and responsibilities by professional nurses
3. Future researchers should adopt a more objective determination of adherence among professional and auxiliary nurses since this study was limited by self-ratings by the staff which could have exposed the study to social-desirability responses
4. Finally, staffing norms and ward management strategies should be reconsidered to maximize the existing limited numbers of staff on the ward to promote efficiency.

## Additional file

Additional file 1: Questionnaire. List of questions in Questionnaire. (PDF 53 kb)

## Abbreviations

CPD: Continuous professional development
GHS: Ghana Health Service
IHR: Institute of Health Research
NG: Nasogastric
NGTF: Nasogastric tube feeding
NMC: Nursing and Midwifery Council
OLR: Ordered Logistic Regression
OR: Odds ratio
REC: Research Ethics Committee
SD: Standard deviation
SOPs: Standard operating procedures
UHAS: University of Health and Allied Sciences
VIF: Variance Inflation Factor
WHO: World Health Organization

## Footnotes

1 S= $\frac{X^2 NP\left(1-P\right)}{d^2\left(N-1\right)+{X}^2P\left(1-P\right)}$, where: *X*^2^= the table value of chi-square for 1 degree of freedom at the desired confidence level N = the population size (166); P = the population proportion (assumed to be 50% since this would provide the maximum sample size); D = the degree of accuracy expressed as a proportion
