# Supplementary material for: Adherence to standard nursing protocols on nasogastric tube feeding in a secondary referral hospital in Ghana: comparing self-ratings by professional and auxiliary nurses
Source: BMC Health Serv Res. 2019 Feb 13;19:119. doi: 10.1186/s12913-019-3931-6 (PMC6375159; doi:10.1186/s12913-019-3931-6)
Supplement: Supplementary file 1 — Questionnaire. List of questions in Questionnaire. (PDF 53 kb) [file 12913_2019_3931_MOESM1_ESM.pdf]

UNIVERSITY OF HEALTH AND ALLIED SCIENCES, HO
SCHOOL OF NURSING AND MDIWIFERY

Research Topic: Adherence to standard nursing protocols on nasogastric tube feeding in a secondary referral hospital in Ghana: comparing self-ratings by professional and auxiliary nurses.

Preamble

This is a questionnaire developed by students of University of Health and Allied Sciences on the research topic "Adherence to standard nursing protocols on nasogastric tube feeding in a secondary referral hospital in Ghana: comparing self-ratings by professional and auxiliary nurses". Your responses are confidential and none of your information will be communicated or shared with a third party without your prior notice and permission. Thank you.

**PART A: SOCIO-DEMOGRAPHIC CHARACTERISTICS OF RESPONDENTS**	
**QUESTIONS**	**RESPONSES**
1. Gender	Male [ ]<br>Female [ ]
2. Age	……………………
3. Religion	Christian [ ]<br>Muslim [ ]<br>Others [ ]
4. Marital Status	Single [ ]<br>Married [ ]<br>Others [ ]
5. Educational Level	Certificate [ ]<br>Diploma [ ]<br>Degree [ ]<br>Masters [ ]<br>PhD [ ]
**6.** Years of Working Experience	……………………
7. Professional Category	Auxiliary Nurses [ ]<br>Registered General Nurses [ ]

**PART B: KNOWLEDGE AND PRACTICE LEVEL OF NURSES**		
**QUESTIONS**	**RESPONSE**	**CODE**
1. Introduction and explaining of procedure is to pre-inform the client	Strongly agree……………………………1<br>Agree…………………………………….2<br>Undecided………………………………..3<br>Disagree…………………………………..4	
2. Upright position during intubation of patients is done to prevent aspiration	Strongly agree……………………………1<br>Agree……………………………………..2<br>Undecided………………………………..3<br>Disagree…………………………………4	

3. Lubrication of the tip of the tube is done for easy passage and prevention of lacerations of the GIT lining	Strongly agree……………………………1<br>Agree………………………………......2<br>Undecided……………………………...3<br>Disagree………………………………..4	
4. Patient mouth is checked while passing tube to observe coiling of the tube in the mouth	Strongly agree……………………………1<br>Agree……………………………..........2<br>Undecided……………………………..3<br>Disagree………………………………..4	
5. Aspirating of the gastric content is a confirmatory method used to check for the position of tube.	Strongly agree……………………………1<br>Agree…………………………………...2<br>Undecided…………………..……….3<br>Disagree………………………………..4	
6. Kinking tube while feeding is done by pinching	Strongly agree……………………………1<br>Agree…………………………………...2<br>Undecided……………...………………3<br>Disagree………………………………..4	
7. Amount of water used to flush the tube before and after feeding is 10mls-15mls	Strongly agree……………………………1<br>Agree…………………………………...2<br>Undecided………..……………………3<br>Disagree………………………………..4	
8. Checking the temperature of the feed is done to prevent irritation of the GIT	Strongly agree……………………………1<br>Agree…………………………………...2<br>Undecided…..…………………………3<br>Disagree………………………………..4	
9. The tube is spigot immediately after feeding to prevent abdominal distention	Strongly agree……………………………1<br>Agree…………………………………...2<br>Undecided……………………………...3<br>Disagree………………………………..4	
10. The syringe for feeding is raised to allow the feed run slowly under gravity	Strongly agree……………………………1<br>Agree………………………………….2<br>Undecided…..…………………………3<br>Disagree………………………………..4	
**PART C: BARRIERS TO STANDARD PRACTICE**		
**QUESTONS**	**RESPONSE**	**CODE**
1. Is there a general standard protocol for NG tube feeding?	Yes ………………………………..1<br>No ……………………………………2<br>If YES, please answer question 2.	
2. Is the protocol available on the ward?	Yes ……………………………………1<br>No ……………………………………..2	
3. Are there adequate number of staff on a shift?	Yes ……………………………………1<br>No ……………………………………..2	

4. If NO to question 3, to what extent does it affect the management of patient on NGT	Very large extent…………………………1<br>Large extent……………………………….2<br>Undecided……….……………….………..3<br>Little extent………..……………………..4	
5. Are there adequate number of NG tubes on the ward?	Yes ……………………………………1<br>No …………………………………….2	
6. If NO to question 5, to what extent does it affect the management of patient on NGT?	Very large extent…………………………1<br>Large extent……………………………….2<br>Undecided……….……………….………..3<br>Little extent………..……………………..4	
7. Do relatives oppose the use of NG tube?	Yes ……………………………………1<br>No …………………………………….2	
8. If YES to question 7, to what extent does it affect the management of patient on NGT?	Very large extent…………………………1<br>Large extent……………………………….2<br>Undecided……….……………….………..3<br>Little extent………..…………………….…4	
9. Have you ever nursed a patient on NG tube in the last six months	Yes ……………………………………1<br>No …………………………………….2	
10. Have you attended a workshop on the management of patient on NG tube in the last six months?	Yes ……………………………………1<br>No …………………………………….2	
11. If YES to question 10, who organized the workshop?	The Hospital…………………………..…1<br>NGO…………………………………..……2<br>Others (please specify)……………………3	
12. Have you had any continuous education on the management of patient on NG tube in the last one year?	Yes ……………………………………1<br>No …………………………………….2	

THANK YOU FOR YOUR TIME
